# Social inequalities in return-to-work among colorectal cancer survivors in Germany

**DOI:** 10.1007/s00520-026-10827-3

**Published:** 2026-06-12

**Authors:** Johannes Soff, Ibrahim Demirer, Nora Tabea Sibert, Clara Breidenbach, Nicole Ernstmann, Paula Heidkamp, Lina Heier, Kati Hiltrop, Sophie Klara Schellack, Johanna Weiß, Oliver Rick, Stefan Rolf Benz, Nico Dragano, Christoph Kowalski

**Affiliations:** 1 Department of Health Services Research, German Cancer Society, Kuno-Fischer-Straße 8, 14057 Berlin, Germany; 2 Institute of Medical Sociology, Centre for Health and Society, Medical Faculty and University Hospital, Heinrich Heine University Düsseldorf, Düsseldorf, Germany; 3 Department for Psychosomatic Medicine and Psychotherapy, Center for Health Communication and Health Services Research, University Hospital Bonn, Bonn, Germany; 4 Department of Research Methods, Faculty of Human Sciences, University of Cologne, Cologne, Germany; 5 Oncological Health Services Research, Clinic for Gynaecology and Obstetrics, University Hospital Düsseldorf, Düsseldorf, Germany; 6 Center for Digital Medicine, Heinrich-Heine University Düsseldorf, Düsseldorf, Germany; 7 Centre for Integrated Oncology Aachen, Bonn, Cologne, Düsseldorf (CIO ABCD), Düsseldorf, Germany; 8 Faculty of Medicine and University Hospital Cologne, Institute of Medical Sociology, Health Services Research and Rehabilitation Science, Chair of Health Services Research, University of Cologne, Cologne, Germany; 9 Department of Clinical Pharmacy and Toxicology, Maastricht University Medical Center, Maastricht, The Netherlands; 10 CARIM School for Cardiovascular Disease, Maastricht University, Maastricht, The Netherlands; 11 Clinic Reinhardshoehe, Center for Oncology Rehabilitation, Bad Wildungen, Germany; 12 Department for Abdominal and Pediatric Surgery, Klinikverbund-Suedwest, Klinken Böblingen, Böblingen, Germany

**Keywords:** Return to work, Cancer, Socio-economic indicators, Social determinants of health

## Abstract

**Background:**

For working-age patients with colorectal cancer, returning to work is a key rehabilitation goal. This study examines the association between socio-economic position (SEP) and return-to-work (RTW), including competing outcomes (pension entry and death), following medical rehabilitation.

**Methods:**

German Pension Insurance data (2013–2022) were analysed, including 32,174 formerly employed patients (ICD-10 C18–C20) of working age undergoing oncological rehabilitation. Patients were categorised by SEP (education, occupational position, income). Logistic regression and restricted mean survival time (RMST) analyses investigated socio-economic differences in RTW, unemployment, sick leave, pension, and death.

**Results:**

Following rehabilitation, 31% of patients returned to work in the first month, and by 2 years, 65% had experienced a long-term RTW lasting ≥ 6 months. SEP was significantly associated with both initial and long-term RTW. The strongest associations were observed for income (long-term RTW highest vs. lowest income tertile: adjusted odds ratio [aOR] 3.06; 95% CI 2.84–3.31) and university education (long-term RTW ISCED‑2011 levels 5–8 vs. 1–3: aOR 2.20; 95% CI 1.94–2.50). RMST showed that patients with higher SEP spent more time in employment and less time unemployed or receiving disability pension than those with lower SEP. The employment time lost between the highest and lowest SEP levels was 8 months for education, 8 months for occupational position, and 10 months for income.

**Conclusions:**

Fifty-eight percent of patients with colorectal cancer were stably employed 2 years after medical rehabilitation. However, despite universal access to rehabilitation in Germany, strong social inequalities in RTW were observed. Monthly employment and pension data allowed reliable estimates, preventing selective non-response and recall bias. However, the results might not generalise to those who do not undergo rehabilitation.

**Supplementary Information:**

The online version contains supplementary material available at 10.1007/s00520-026-10827-3.

## Introduction

Colorectal cancer is the third most common cancer diagnosis for men and the second most common for women both worldwide and in Germany [1, 2]. In Germany, approximately 55,000 new cases are diagnosed annually, with a relative 5-year survival rate of 64% [1]. A considerable number of patients (approximately 30%) receive the diagnosis at working age with consequences for future employment [3]. In particular, physical functional limitations and sometimes persistent symptoms due to illness and prolonged periods of therapy can restrict the patient’s employment, earnings, and other related role activities [4, 5]. This off-work period can range from a temporary interruption to a longer incapacity to work, a voluntary or involuntary change of job, or a complete loss of income [6]. Overall, cancer survivors are 1.4 times more likely to be unemployed compared to healthy controls and have an increased risk of early retirement [7]. Gastrointestinal tumours and their treatment (e.g. a stoma) are associated with an additional increased risk of long-term sickness absence, unemployment, and job loss compared with other types of cancer [8, 9]. Although not all patients wish to return to work, returning is generally considered an important rehabilitation goal and often signifies a milestone in restoring a structure of daily living that plays an important role in the patient’s sense of normality and overall well-being [10]. At the same time, work-related decisions after cancer diagnosis are not solely determined by health factors, but are also shaped by individual preferences and behavioural factors [11]. These include the personal value attributed to work, as well as the job requirements under which a return-to-work (RTW) is considered feasible [12]. These requirements may differ depending on socio-economic characteristics [13]. For patients in paid employment at the time of diagnosis, a timely and stable RTW is particularly crucial [14].

Questionnaire-based studies on RTW after colorectal cancer treatment [15–20] show varying RTW rates, ranging from 39 to 89% depending on the setting and study design. Studies showing higher RTW rates also report lower response rates, suggesting an overestimation of the RTW results. Register-based studies from the Netherlands, Denmark, and Sweden estimate RTW rates between 37 and 67% within 1 to 2 years after diagnosis [21–23].

Differences in RTW can arise from inequalities at the macro, meso, and micro level. At the macro level, the health and social-care system influences RTW regulations and possibilities for cancer survivors [24]. The macro level influences the meso level by shaping the social context in which the RTW takes place, including partner relationships, family life, work environments, and other public settings [25]. On the micro level, variations in RTW rates cannot be attributed solely to differences in medical factors such as tumour stage or treatments. Rather, they are presumably also related to other patient characteristics such as their socio**-**economic position (SEP) or occupational type [25, 26]. Employment difficulties and the financial problems that accompany them can vary between groups of different SEPs. Educational inequalities lead to differences in access to occupations and employment opportunities, which also influence inequalities in health risks and resources such as income [27]. This socially graded pattern is well studied in the general rehabilitative context, with oncology patients often having the least favourable rehabilitation outcomes [28]. Yet, the analysis of socio**-**economic factors among colorectal cancer survivors has shown heterogeneous effects on the RTW [29, 30]. A systematic review on prognostic factors of RTW has found inconclusive evidence regarding the association between both education and occupation and RTW [30]. So far, the existing research on this topic often relies on small samples and survey data, which is prone to underestimating SEP differences, as non-participation in population studies is likely to be a source of bias [31–33].

Here we examined the association between various socio-economic indicators (education, income, occupation) and the RTW among formerly employed patients with colorectal cancer following medical rehabilitation in Germany, using comprehensive routine data from the German Pension Insurance. In addition, the study seeks to describe their long-term employment trajectory up to 5 years post-rehabilitation, accounting for time-varying states and the occurrence of competing events such as pension episodes and death.

## Methods

### Data basis

In this study, claims data from the German Pension Insurance on completed rehabilitation measures from 2013 to 2022 were used [34]. The dataset is based on the German rehabilitation statistics database and contains person-based information from various data sources. These sources include the insurance account (information from the reporting procedure between employers and the social insurance, e.g. employment information), the rehabilitation application (self-reported, e.g. marital status), and the medical discharge report (rehabilitation physician-reported, e.g. work ability at discharge from medical rehabilitation). Detailed information on the setting and the resulting sample cohort are further described in the Supplementary Methods. A detailed data description in German can be found in the code plan of the Research Data Centre [35]. The complete data description of the project can be found at the following link: https://osf.io/s9jwq/wiki/Data%20Description/. This study was conducted and reported in line with the STROBE and ESMO-GROW guidelines [36, 37].

### Sample

For the analysis, we included all patients aged 18 to 62 with at least one oncological rehabilitation and a primary diagnosis of colorectal cancer (ICD10 C18–20), who reported being employed when they applied for the rehabilitation measure. Patients who already received a pension before the start of their first rehabilitation or had no recorded income subject to social security contributions 2 years prior to the calendar year of medical rehabilitation were excluded.

**Fig. 1:**
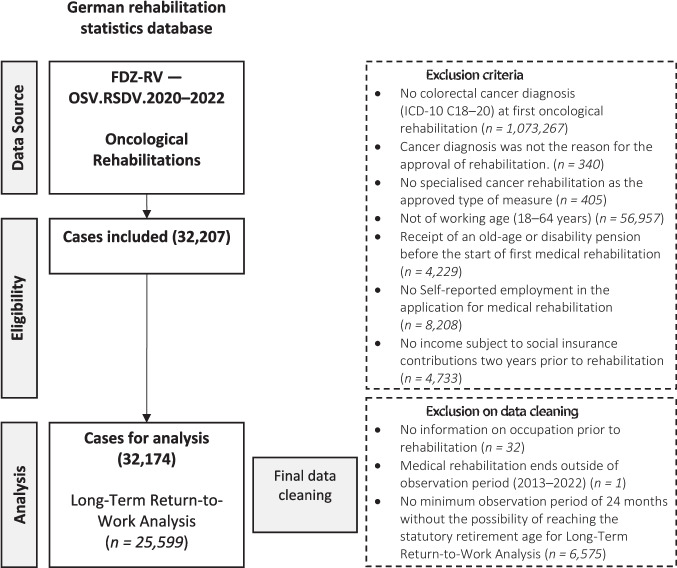
ESMO-GROW flowchart for real-world evidence studies in oncology. The data flow chart illustrates the selection process from the pension insurance database with all patients who completed at least one medical oncological rehabilitation measure in the reporting period to the analysed dataset of all 32,174 previously employed colorectal cancer survivors. Before the analysis in the secure data environment, further exclusion criteria were formulated on a theoretical basis, but these did not lead to any exclusions. These additional criteria are tumour diagnosis not verified, rehabilitation did end prematurely with or without a physician’s consent or for medical reasons, rehabilitation not completed or record incomplete, death during medical rehabilitation. Abbreviations: ICD-10, 10th revision of the International Statistical Classification of Diseases and Related Health Problems

Study entry time was determined by the start date of the first medical rehabilitation episode, which could have been any date between January 1, 2013, and November 30, 2022. The age limit of 64 years at the end of medical rehabilitation aligns with the European Union’s employment age [38] and prevents receiving an old-age pension within 2 years after rehabilitation ends. Those for whom this did not apply because they could reach the statutory retirement age at an age younger than 66 (born before 1958) were excluded from analyses requiring 2 years of observation (long-term RTW). Therefore, all patients included in the analysis had the opportunity to experience the measured RTW endpoints. A flowchart of the sample selection can be found in Fig. 1.

### Indicators of socio-economic position

In line with prior SEP taxonomy [28], the following three indicators were used: education, income, and occupational position. Education was classified according to the International Standard Classification of Education (ISCED-2011) of the UNESCO [39], into three categories: (1) primary or secondary education (ISCED-2011 levels 1–3), (2) post-secondary non-tertiary education (ISCED-2011 level 4), or (3) tertiary education (ISCED-2011 levels 5–8). Income 2 years prior to the calendar year of medical rehabilitation was reported in the categories: (1) low (1st to 33rd percentile), (2) medium (34th to 66th percentile), and (3) high (67th to 100th percentile). These reflect assessable earnings subject to social security contributions, truncated at the contribution assessment ceiling (income threshold up to which insured persons’ contributory earnings are taken into account for the calculation of statutory social security contributions). Table S1 shows the income thresholds for the years in the observation period. Information on the most recently reported occupational positions was categorised into distinct groups based on the Blossfeld occupational classification [40]: (1) unskilled, manual (occupations with a high proportion of unskilled workers, ≥ 60%, e.g. labourers, assembly workers); (2) skilled, manual (occupations with a lower proportion of unskilled workers, ≤ 40%, e.g. electricians, mechanical engineers); (3) unskilled, non-manual (simple commercial and administrative occupations, e.g. basic clerical and retail roles); (4) skilled, non-manual (qualified commercial and administrative occupations with intermediate to higher administrative or distributive tasks, e.g. certain service, or scientifically oriented roles); and (5) highly skilled (occupations requiring the ability to solve scientific or technical problems, or involving significant managerial control and decision-making authority, e.g. engineers, managers, and senior officials). Information on educational level and occupational position was based on the annual notification from employer to the German Pension Insurance. Missing values of the education variable were integrated as a distinct category (“Unknown”) into the analysis.

### Return to work and its competing events

RTW was defined on the basis of the ICF (International Classification of Functioning, Disability and Health) category d850 “Remunerative employment”, which encompasses all aspects of work, as an occupation, trade, profession, or other form of paid employment subject to social security contributions [41]. Employment was measured using the patient’s pension insurance contribution history, which provides details of the employment status for each month. To reflect the different phases of the rehabilitation process, RTW was operationalised in different endpoints based on the conceptualisation of RTW by Young et al. [42]. The “Initial RTW” was defined as the immediate transition from inpatient treatment to employment in the first month after completion of medical rehabilitation (phase 2/Re-Entry after Young et al.). “Long-Term RTW” was considered successful if a person was employed for at least 6 consecutive months within 2 years following medical rehabilitation (phase 3/Maintenance after Young et al.). The 2-year timeframe ensured that patients who received the maximum duration of sickness benefits (in Germany, 78 weeks) had the theoretical possibility of experiencing a “Long-Term RTW”.

**Fig. 2:**
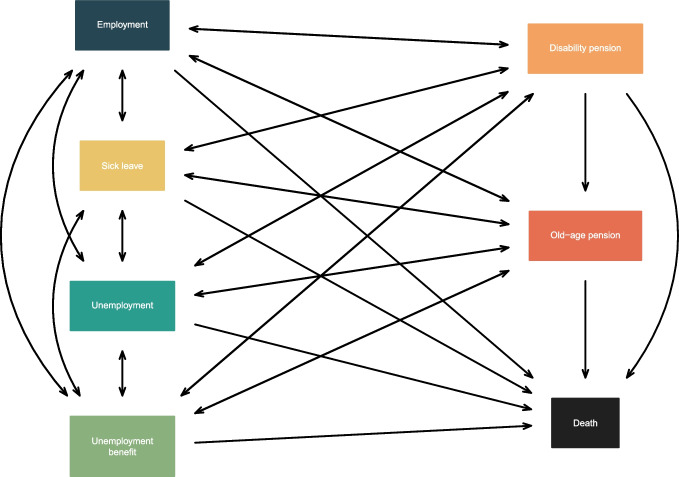
Graphical presentation of the restricted mean survival time model with all observed states (boxes) and transitions (arrows). The figure illustrates various labour market and social security transitions, including employment, sick leave, unemployment benefit, unemployment, disability pension, old-age pension, and death. Categories reflect mutually exclusive statuses at a given time point. A detailed description of the observed states is presented in Table S2. Flowchart depicting transitions between employment, sick leave, unemployment benefit, and unemployment, leading to disability pension, old-age pension, and death

To describe competing events of employment and differentiate non-employment episodes (phase 1/Off Work after Young et al.), the following states were analysed additionally: unemployment, unemployment benefit, sick leave (work absence), disability pension, old-age pension, and death. The possible transitions between states in the model are shown in Fig. 2. A description of each observed state is provided in Table S2. All endpoints are distinct events that are not mutually exclusive over time. Patients may experience several endpoints in succession during the observation period. Yet, in event-time analyses, the states at any given point in time are mutually exclusive as employment status, sick leave, unemployment, and unemployment benefit are based on an aggregated variable, which assigns one state per month per person. The patients were followed up for up to 5 years until December 31, 2022, for all specified endpoints.

### Statistical analysis

Descriptive statistics were used to describe the sample characteristics and outcomes. Restricted mean survival time (RMST) [43] was calculated for the 5-year observation period to investigate the association of each socio**-**economic indicator on the state probability for all outcomes. RMST was used instead of traditional Cox proportional hazard models because it does not require the proportional hazard assumption to be met and allows the outcomes to be expressed as the absolute difference in the average number of months in the corresponding state. Individuals were only censored at the end of the follow-up period. For the adjusted analysis, logistic regressions were performed for initial and long-term RTW, accounting for potential confounding in the association between SEP and RTW. The minimum adjustment set for confounding was identified in a directed acyclic graph [44] (Figure S1). All estimators were adjusted for the confounders age, sex and citizenship. Occupational position was also adjusted for education. Income was additionally adjusted for education and occupational position. All analyses were performed using R 4.5.0 [45] and primarily the software packages tidyverse [46], survival [47], and performance [48] for creating, testing, and visualising the models.

## Results

### Sample characteristics

After applying the selection criteria, 32,174 patients with a primary cancer of the colon or rectum remained in the final data set. The majority had post-secondary education (71%). In total, 72% of patients were in non-manual occupations, and 62% were skilled or highly skilled. The sample included a higher proportion of malignant neoplasms of the colon (55%) than of the rectum (41%), as well as a higher proportion of men (61%) than women (39%). Further details of the study population are shown in Table 1.

**Table 1:** Baseline characteristics of 32,174 patients with colorectal cancer undergoing medical rehabilitation in Germany, 2012–2022

**Characteristics**	**Male** *N* = 19,689	**Female** *N* = 12,485
**Age, Median (IQR)**	56 (52–60)	55 (51–59)
**Marital status, *n* (%)**		
Married	15,102 (77)	8517 (68)
Single	2582 (13)	1479 (12)
Divorced	1543 (7.8)	1663 (13)
Widowed	223 (1.1)	479 (3.8)
Not recorded	239 (1.2)	347 (2.8)
**Citizenship, *n* (%)**		
Germany	18,528 (94)	11,894 (95)
Italy, Spain, Greece, Portugal	239 (1.2)	97 (0.8)
Former Yugoslavia	184 (0.9)	109 (0.9)
Republic of Türkiye	253 (1.3)	53 (0.4)
Other	485 (2.5)	332 (2.7)
**Education, *n* (%)**		
Primary or secondary (ISCED-2011: 1–3)	1588 (8.1)	1230 (9.9)
Post-secondary (ISCED-2011: 4)	13,941 (71)	8844 (71)
Tertiary (ISCED-2011: 5–8)	2192 (11)	1371 (11)
Unknown	1968 (10.0)	1040 (8.3)
**Occupational position, *n* (%)**		
Unskilled, manual	2389 (12)	504 (4.0)
Skilled, manual	5363 (27)	714 (5.7)
Unskilled, non-manual	5795 (29)	3493 (28)
Skilled, non-manual	3895 (20)	6818 (55)
Highly skilled	2247 (11)	956 (7.7)
**Income, *n* (%)**^**a**^		
Low	4359 (22)	6369 (51)
Middle	7045 (36)	3680 (29)
High	8285 (42)	2436 (20)
**Employment status when applying for medical rehabilitation, *n* (%)**		
Full-time	19,208 (98)	8058 (65)
Part-time	481 (2.4)	4427 (35)
**Sickness absence within the last 12 months before medical rehabilitation, *n* (%)**		
< 3 months	5801 (29)	3846 (31)
3–6 months	3123 (16)	1994 (16)
> 6 months	10,765 (55)	6645 (53)
**Physician-reported work ability at discharge from medical rehabilitation, *n* (%)**		
Able to work	2554 (13)	1591 (13)
Unable to work	17,048 (87)	10,836 (87)
No medical assessment	87 (0.4)	58 (0.5)
**Primary diagnosis, *n* (%)**		
Malignant neoplasm of the colon (ICD-10 C18)	10,229 (52)	7439 (60)
Malignant neoplasm of the rectosigmoid junction (ICD-10 C19)	787 (4.0)	524 (4.2)
Malignant neoplasm of the rectum (ICD-10 C20)	8673 (44)	4522 (36)
**Number of comorbidities, *n* (%)**		
No comorbidities	4494 (23)	3005 (24)
1–2 comorbidities	9849 (50)	6409 (51)
≥ 3 comorbidities	5346 (27)	3071 (25)

^a^The income tertiles are based on the annual income subject to social insurance contributions two years prior to medical rehabilitation. The income thresholds for 2020 were low income below €26,940, middle income between €26,940.01 and €43,458, and high income above €43,458

*ICD-10*, 10th revision of the International Statistical Classification of Diseases and Related Health Problems; *ISCED 2011*, International Standard Classification of Education—2011 version

Data source: FDZ-RV—OSV.RSDV.2020–2022

### Outcome frequencies and restricted mean time

Overall, 31% of the patients initially returned to work after medical rehabilitation, and 65% of patients had a long-term RTW. During the observation period, 17% received unemployment benefits for at least one month, 21% started receiving an old-age pension, 24% started receiving a disability pension, and 14% of patients died. For all three SEP indicators, patients with higher positions had a higher proportion of successful RTW. The distribution of all outcomes, stratified by the three indicators of SEP, is presented in Table 2.

**Table 2:** Absolute and relative frequency of work, pension, and mortality related outcomes of 32,174 colorectal cancer patients in Germany, 2013–2022

Indicators of the socio-economic position	Successful initial RTW	Successful long-term RTW	Unemployment benefit receipt	Disability pension	Old-age pension	Death
**Education, *n* (%)**						
Primary or secondary (ISCED-2011: 1–3)	718 (25)	1361 (61)	624 (22)	752 (27)	632 (22)	375 (13)
Post-secondary (ISCED-2011: 4)	6882 (30)	11,793 (65)	3860 (17)	5718 (25)	4604 (20)	3063 (13)
Tertiary (ISCED-2011: 5–8)	1645 (46)	2143 (78)	468 (13)	469 (13)	766 (21)	443 (12)
Unknown	707 (24)	1278 (54)	656 (22)	828 (28)	699 (23)	479 (16)
**Occupational position, *n* (%)**						
Unskilled, manual	684 (24)	1399 (60)	567 (20)	821 (28)	649 (22)	438 (15)
Skilled, manual	1721 (28)	3063 (63)	1051 (17)	1513 (25)	1356 (22)	917 (15)
Unskilled, non-manual	2236 (24)	4282 (58)	2029 (22)	2606 (28)	1978 (21)	1231 (13)
Skilled, non-manual	3853 (36)	5912 (69)	1515 (14)	2342 (22)	2091 (20)	1370 (13)
Highly skilled	1458 (46)	1919 (76)	446 (14)	485 (15)	627 (20)	404 (13)
**Income, *n* (%)**						
Low	2519 (23)	4520 (53)	2485 (23)	3380 (32)	2184 (20)	1482 (14)
Middle	2885 (27)	5504 (65)	1819 (17)	2689 (25)	2319 (22)	1440 (13)
High	4548 (42)	6551 (77)	1304 (12)	1698 (16)	2198 (21)	1438 (13)
**Total, *n* (%)**	9952 (31)	16,575 (65)	5608 (17)	7767 (24)	6701 (21)	4360 (14)

The table shows the absolute and relative frequencies of the dichotomous endpoints for each level of the socio-economic indicators for the study sample. The endpoints are distinct events. Patients can experience several endpoints in succession. Initial RTW—employment in the first month after the medical rehabilitation. Long-term RTW—employment of ≥ 6 months in the 2 years after the rehabilitation. Therefore, long-term RTW was calculated only for individuals who had been observed for at least two years and who would not reach statutory retirement age within this timeframe (*n* = 25,599). In order to have a successful long-term RTW, an initial RTW is not necessarily required. Disability pension includes full and partial pensions. In this sample, 99% (*n* = 7696) of recipients were eligible for full disability pension (unable to work > 3 h/day in any occupation)

*ISCED 2011*, International Standard Classification of Education—2011 version; *RTW*, return-to-work

Data source: FDZ-RV—OSV.RSDV.2020–2022

**Fig. 3:**
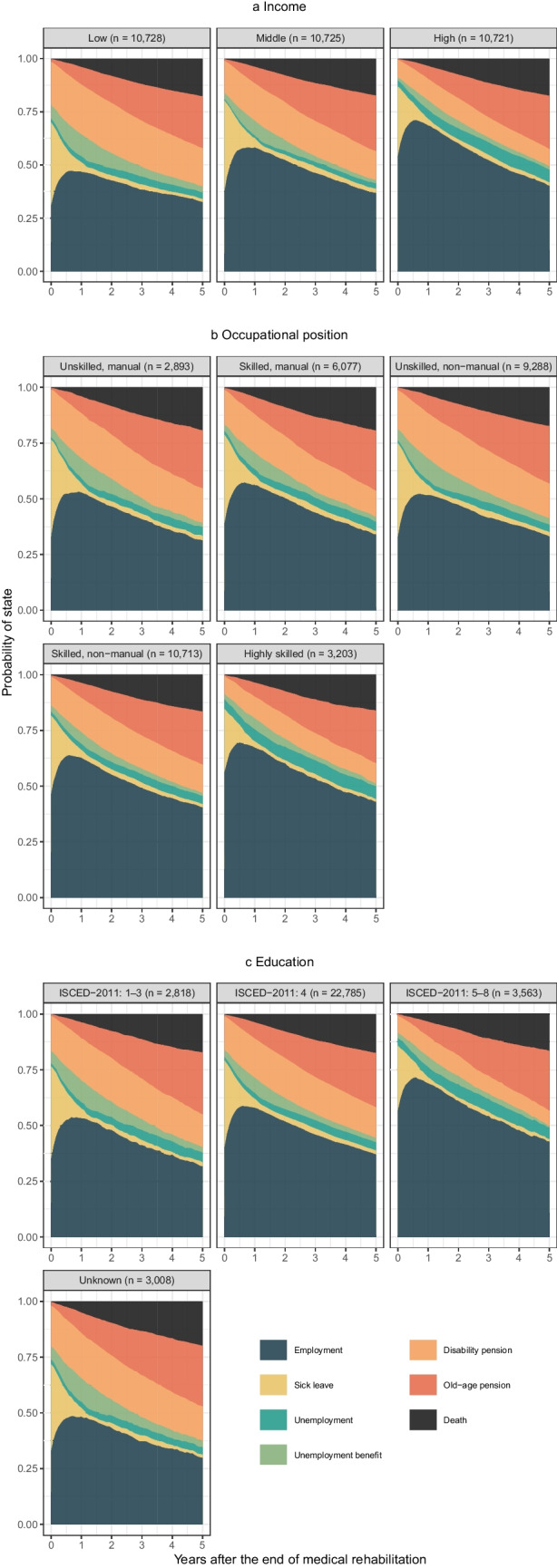
Comparison of the probabilities of employment, unemployment, unemployment benefit, sick leave, disability pension, old-age pension, and death after medical rehabilitation, stratified by income (**a**), occupational position (**b**), and education (**c**) among 32,174 patients with colorectal cancer in Germany, 2013–2022. The figure shows the differences in the estimated average number of months for the states “employment”, “unemployment”, “unemployment benefit”, “sick leave”, “disability pension”, “old-age pension”, and “death” over a period of 5 years after the end of medical rehabilitation. Table S3 shows the restricted mean survival time for each state in months over the observation period of 5 years. Abbreviations: *ISCED 2011*, International Standard Classification of Education—2011 version. Data source: FDZ-RV—OSV.RSDV.2020–2022. Graphs comparing the probabilities of being in a state for different levels of the socio-economic indicators, with higher probabilities of being employed and lower probabilities of being unemployed or in disability pension for patients with a higher socio-economic position

The descriptive results were expanded by the RMST analyses (Fig. 3). After medical rehabilitation, people with tertiary education spent on average 8 months more of the following 5 years in employment than people with primary or secondary education and 5 months more than people with post-secondary education. Correspondingly, the higher educated spent less time in disability pension. A similar pattern is observed for income, with the low-income group spending the least time in employment (2 years) and the high-income group the most time (2 years and 10 months). The RMSTs according to occupational position were less consistent. The group of unskilled workers (regardless of whether they were in manual or non-manual occupations) spent the least time (2 years and 2 months) in employment and the longest time on the disability pension (11 months). Skilled manual workers spent 2 months more in employment and 2 months less each in disability pension. The highest skilled workers started work earlier after rehabilitation (2 months of sick leave) and retired later (7 months of old-age pension, 6 months of disability pension) than the other groups, thus remaining in employment for more than half of the 5 years. Table S3 shows all RMSTs of 5 years reported in months. Figure S2 shows the RMSTs for male and female patients separately. Similar patterns were observed in total time spent in different states, although the distribution among individual indicators differed by sex. The sex difference was most pronounced for the low-income category, where female patients spent 5 months longer in employment than male patients. The sex-specific RMST for the 5 years is presented in Tables S4 and S5.

### Multivariate results of logistic regressions

Table 3 displays the unadjusted (OR) and adjusted odds ratios (aOR) of RTW according to the SEP (for the performance plots cf. Figures S3 and S4). Social gradients are consistent and statistically significant in both the unadjusted and the adjusted models for tertiary education, skilled, non-manual occupations, and income differences. The income indicator showed the strongest association. This was particularly pronounced in the long-term RTW of the group with a high income compared to the group with a low income (aOR 3.06; 95% CI 2.84–3.31). Those in skilled manual occupations had 21% higher odds of an initial RTW compared to those in unskilled manual occupations. However, there was no significant difference in long-term RTW between the groups. The group with missing information on education had the lowest odds of both initial and long-term RTW. The associations of the sex-specific analyses are shown in Tables S6 and S7 along with their performance plots in Figures S5–S8. These show that the socio-economic differences are more pronounced for all three indicators among male patients compared to female patients. Sex-specific differences were observed between low- and middle-income groups in the initial RTW. The OR was 46% lower for male workers in the lower-income group compared to those in the middle-income group. No significant differences in the initial RTW were observed between the low- and middle-income groups of female workers.

**Table 3:** Association between socio-economic position and return-to-work of 32,174 colorectal cancer patients in Germany, 2012–2022

Indicators of socio-economic position	Initial RTW			Long-term RTW		
	**Event/*n***	**OR (95% CI)**	**aOR (95% CI)**	**Event/*n***	**OR (95% CI)**	**aOR (95% CI)**
**Education**^***a***^						
Primary or secondary (ISCED-2011: 1–3)	718/2818	—	—	1361/2247	—	—
Post-secondary (ISCED-2011: 4)	6882/22,785	1.27 (1.16 to 1.38)	1.20 (1.10 to 1.32)	11,793/18,236	1.19 (1.09 to 1.30)	1.13 (1.03 to 1.24)
Tertiary (ISCED-2011: 5–8)	1645/3563	2.51 (2.25 to 2.79)	2.37 (2.13 to 2.64)	2143/2742	2.33 (2.06 to 2.64)	2.20 (1.94 to 2.50)
Unknown	707/3008	0.90 (0.80 to 1.01)	0.87 (0.78 to 0.99)	1278/2374	0.76 (0.68 to 0.85)	0.73 (0.65 to 0.82)
**Occupational position**^***a,b***^						
Unskilled, manual	684/2893	—	—	1399/2347	—	—
Skilled, manual	1721/6077	1.28 (1.15 to 1.41)	1.21 (1.09 to 1.34)	3063/4893	1.13 (1.03 to 1.25)	1.06 (0.96 to 1.18)
Unskilled, non-manual	2236/9288	1.02 (0.93 to 1.13)	1.04 (0.95 to 1.15)	4282/7330	0.95 (0.87 to 1.05)	0.96 (0.87 to 1.05)
Skilled, non-manual	3853/10,713	1.81 (1.65 to 1.99)	1.77 (1.60 to 1.95)	5912/8515	1.54 (1.40 to 1.69)	1.44 (1.30 to 1.60)
Highly skilled	1458/3203	2.70 (2.42 to 3.01)	2.21 (1.97 to 2.48)	1919/2514	2.19 (1.93 to 2.47)	1.69 (1.48 to 1.94)
**Income**^***a,b,c***^						
Low	2519/10,728	—	—	4520/8556	—	—
Middle	2885/10,725	1.20 (1.13 to 1.28)	1.20 (1.12 to 1.28)	5504/8520	1.63 (1.53 to 1.73)	1.77 (1.65 to 1.89)
High	4548/10,721	2.40 (2.26 to 2.55)	2.08 (1.94 to 2.22)	6551/8523	2.97 (2.78 to 3.17)	3.06 (2.84 to 3.31)

^*a*^Adjusted for sex, age and citizenship

^*b*^Additionally adjusted for education

^*c*^Additionally adjusted for occupational position

*CI* confidence interval, *OR *odds ratio, *aOR *adjusted odds ratio, *ISCED 2011* International Standard Classification of Education—2011 version, *RTW* return-to-work

The models for initial RTW are based on the complete study sample of 32,174 colorectal cancer patients. The models for long-term RTW require a minimum observation period of 24 months, during which the statutory retirement age is not reached. These models are therefore based on 25,599 colorectal cancer patients

Em dash (—) indicates the reference category

Data source: FDZ-RV—OSV.RSDV.2020–2022

## Discussion

This study investigated the associations between indicators of SEP and RTW among colorectal cancer patients undergoing rehabilitation in Germany. These associations followed a social gradient with higher probabilities of RTW and employment among those in higher positions. This gradient was more pronounced among male than female patients. For income and education, ORs were consistent for both endpoints, but less so for occupation. The typical difference between blue-collar and white-collar workers was not observed during the first transition from inpatient treatment to employment. Long-term RTW as an indicator of stable employment revealed differences between qualification levels (skilled vs. unskilled) and between manual and non-manual work. Persistent physical impairments following cancer treatment can limit work ability, particularly with regard to manual tasks. This may explain the lower odds of RTW for more physically demanding occupations. A lower level of professional qualification is associated with a lower level of occupational autonomy and flexibility of work [49]. Therefore, people in unskilled occupations may have fewer opportunities to return-to-work. Additionally, unskilled workers may not have the opportunity to gradually resume their work tasks due to less flexibility in working conditions, a key occupational factor identified as relevant to a successful RTW [49].

The social gradient could be steeper when considering the entire German population, given that individuals with higher incomes (above €82,800 in West Germany in 2020) have the option of taking out private pension insurance and are therefore underrepresented in the German Federal Pension Insurance. The groups with by far the least favourable results, regardless of the statistical method, were the groups with no information on education. Previous analyses showed that people for whom there is no notification from the employer to the insurance providers are socially disadvantaged in numerous health-related outcomes [28] and presumably belong to a group of precariously employed people.

To our knowledge, this is the first study to analyse social inequalities in longitudinal RTW, taking into account competing events in a large sample of formerly employed colorectal cancer patients after medical rehabilitation. All three indicators measure a specific aspect of the SEP at an individual level and build on each other at different stages over the course of a person’s working life [50]. Education represents a broader concept of a knowledge-related asset, directly influences health literacy, and tends to reflect the long-term effects of early life circumstances [51]. Occupational position provides further detail on work requirements. Income provides further insight into the current position and possibly reflects the necessity, flexibility, and voluntariness of paid labour. The sex-specific analyses show a steeper social gradient for men than for women. This can be attributed to the social structures of men and women in Germany, which differ in relevant aspects such as rates of full- and part-time employment, occupational level, and income [52]. In particular, women in Germany are more likely to be employed in part-time positions. This could facilitate a return to part-time employment following an absence. Such patterns could facilitate a more gradual reintegration into the workforce for women compared to men, who are predominantly employed in full-time positions.

These results expand on findings from existing systematic reviews, which highlighted that only limited studies on occupational and socio-economic factors have been conducted to date [29, 30]. den Bakker et al. (2018) included three studies on education and occupation (manual vs non-manual work) in their meta-analysis. They found inconclusive evidence on RTW, as the studies found opposite effects for education and none found a significant effect of occupation [30]. As yet it is difficult to compare RTW results due to differences in operationalisation and health and social systems across countries, the identified long-term RTW rates appear slightly lower than those of colorectal cancer patients in other European countries [9, 16, 20, 53–56]. This may be explained by the 6 months that must be spent in employment to achieve successful long-term RTW. This comparatively long period emphasises the longevity and maintenance of this RTW phase. In order to enable better comparability of RTW results, a standardised definition and operationalisation of RTW is required [29, 57].

A major strength of the current study is the large study population of a nationally representative sample of persons who underwent medical rehabilitation in a specialised oncological clinic with a follow-up period of up to 5 years. Information on monthly employment and pension status from the pension insurance contribution history made it possible to avoid selective non-response and recall bias. Another strength is the availability of different outcome parameters that allowed for a detailed competing event analysis. This increased the generalisability and representation of various possible states to evaluate the associations of SEP and RTW.

When interpreting the findings, a few limitations should be considered. Using pension, insurance, and rehabilitation data results in a lack of oncology-specific clinical patient information. This gap of knowledge is particularly important, given the possible socio**-**economic gradient in screening, time of diagnosis and treatment and the possible effect heterogeneity of different cancer stages [58, 59]. While cancer stage and treatment act as mediators rather than confounders in the causal pathway between SEP prior to the onset of the disease and RTW, the inability to account for these clinical factors prevents us from determining how much of the observed socio-economic inequality in RTW is directly driven by these disease-related disparities. This limits our understanding of the underlying mechanisms and should be acknowledged when interpreting the results.

A further limitation of pension insurance data is that it only lists periods of employment and income subject to social insurance contributions and therefore does not allow distinguishing full-time and part-time employment in the contribution history. Consequently, other forms of work or changes in working hours post-rehabilitation are not reflected in the data. Given that this study is based on observational data, with baseline at medical rehabilitation, the possibility of confounding by indication must be taken into consideration. Patients who were unable or unwilling to undergo rehabilitation, for example due to their medical condition, are therefore not included in the study. Two studies estimate that 50% of colorectal cancer patients in Germany participate in medical rehabilitation programmes [60, 61]. Although these programmes are routinely approved in Germany and patients who do not participate in rehabilitation are often older or unemployed patients, this selection process also limits the generalisability of the results among the working population. It should also be noted that the rehabilitation of civil servants and self-employed persons is generally covered by a different pension system and is therefore widely excluded from the analysed database (12.7% of the German population [62]). However, employees with temporary or fixed-term contracts are compulsorily insured and therefore represented in the data.

The present study examined separate indicators of SEP, and additionally stratified analyses by sex. However, intersectionality theory suggests that individuals are not defined by a single dimension of social position, but by the simultaneous interaction of multiple identities and structural factors, which may produce unique combinations of advantage or disadvantage that are not captured by additive, single-factor models [63]. To further investigate the relationship of multiple marginalised positions and RTW, future research could apply an intersectional analytical framework to cancer survivors [64]. More research is needed on performance measures in cancer patients after their medical rehabilitation to further investigate the challenges faced by those who wish to return to work. Importantly, our results demonstrate that colorectal cancer patients with a lower SEP achieve the rehabilitation goal of RTW far less frequently. This indicates that, despite the comprehensive nature of the German rehabilitation system, it does not sufficiently address the needs of socio-economically disadvantaged groups. Targeted measures must therefore be implemented to improve RTW outcomes for patients with lower SEP, such as specialised support programmes, enhanced case management, and the expansion of low-threshold information and counselling services on vocational rehabilitation options. In particular, it is necessary to raise awareness among both patients and health professionals about these available resources and to facilitate easier access to support, thereby promoting social equity and more successful reintegration into working life for cancer survivors with fewer structural advantages.

## Supplementary Information

Below is the link to the electronic supplementary material.

ESM 1 (PDF 8.81 MB)

## Data Availability

For the analysis, the Rehabilitation Statistics Database (FDZ-RV — OSV.RSDV.2020–2022) of the German Pension Insurance was used, which is reviewed, anonymised, documented and published for external researchers by the Research Data Centre of the German Pension Insurance in compliance with data protection and social confidentiality. The legal basis for the analysis is in particular § 79 SGB IV in conjunction with the general administrative regulation on statistics in pension insurance, according to which the data must be transmitted in anonymised or pseudonymised form for central reporting. The data underlying this article can be obtained on request from the Research Data Centre of the German Pension Insurance and cannot be shared by the author.
