# Supplementary material for: Social inequalities in return-to-work among colorectal cancer survivors in Germany
Source: Support Care Cancer. 2026 Jun 12;34(7):650. doi: 10.1007/s00520-026-10827-3 (PMC13263287; doi:10.1007/s00520-026-10827-3)
Supplement: Supplementary file 1 — (PDF 8.81 MB) [file 520_2026_10827_MOESM1_ESM.pdf]

Social Inequalities in Return to Work Among Colorectal Cancer Survivors in Germany

Johannes Soff[1,2*], Ibrahim Demirer[3,4], Nora Tabea Sibert[5,1,6,7], Clara Breidenbach[1,8], Nicole Ernstmann[8,3], Paula Heidkamp[8,3], Lina Heier[8,3,10,11], Kati Hiltrop[5,9], Sophie Klara Schellack[1], Johanna Weiß[5,9], Oliver Rick[12], Stefan Rolf Benz[13], Nico Dragano[2], Christoph Kowalski[1]

[1] Department of Health Services Research, German Cancer Society, Berlin, Germany

[2] Institute of Medical Sociology, Centre for Health and Society, Medical Faculty and University Hospital, Heinrich Heine University Düsseldorf, Düsseldorf, Germany

[3] Department for Psychosomatic Medicine and Psychotherapy, Center for Health Communication and Health Services Research, University Hospital Bonn, Bonn, Germany

[4] Department of Research Methods, Faculty of Human Sciences, University of Cologne, Cologne, Germany

[5] Oncological Health Services Research, Clinic for Gynaecology and Obstetrics, University Clinic Düsseldorf, Düsseldorf, Germany

[6] Center for Digital Medicine, Heinrich-Heine University Düsseldorf, Düsseldorf, Germany

[7] Centre for Integrated Oncology Aachen, Bonn, Cologne, Düsseldorf (CIO ABCD), Düsseldorf, Germany

[8] Faculty of Medicine and University Hospital Cologne, Institute of Medical Sociology, Health Services Research and Rehabilitation Science, Chair of Health Services Research, University of Cologne, Cologne, Germany

[9] Department of Clinical Pharmacy and Toxicology, Maastricht University Medical Center, Maastricht, The Netherlands

[10] CARIM School for Cardiovascular Disease, Maastricht University, Maastricht, The Netherlands

[11] Clinic Reinhardshoehe, Center for Oncology Rehabilitation, Bad Wildungen, Germany

[12] Department for Abdominal and Pediatric Surgery, Klinikverbund-Suedwest, Klinken Böblingen, Böblingen, Germany

Corresponding author. Johannes Soff, German Cancer Society, Kuno-Fischer-Straße 8, 14057 Berlin, Germany; ORCID ID: 0000-0003-0515-9013, E-Mail: Johannes.Soff@hhu.de

Supplementary Materials - Index

Supplementary Methods

Detailed information on the setting *pag. 3*

Supplementary Figures and Tables

Table S1: Income thresholds for low, medium, and high income for the years 2011–2020 *pag. 4*

Table S2: Description of RTW-relevant states in German Pension Insurance data. *pag. 5*

Figure S1: Simplified directed acyclic graph. *pag. 6*

Table S3: Restricted mean survival time in months of 32,174 colorectal cancer patients in Germany, 2013–2022. *pag. 7*

Figure S2: Comparison of the probabilities of employment, unemployment, unemployment benefit, sick leave, disability pension, old-age pension and death after medical rehabilitation, stratified by income (a), occupational position (b) and education (c) among 19,689 male and 12,485 female patients with colorectal cancer in Germany, 2013–2022. *pag. 8*

Table S4: Restricted mean survival time in months of 19,689 male colorectal cancer patients in Germany, 2013–2022. *pag. 10*

Table S5: Restricted mean survival time in months of 12,485 female colorectal cancer patients in Germany, 2013–2022. *pag. 11*

Figure S3: Visual check of model assumptions for the adjusted logistic regressions of 'Successful Initial RTW' among 32,174 colorectal cancer patients in Germany, 2012–2022. *pag. 12*

Figure S4: Visual check of model assumptions for the adjusted logistic regressions of 'Successful Long−Term RTW' among 25,599 colorectal cancer patients in Germany, 2012–2022. *pag. 13*

Table S6: Association between socio-economic position and Return-to-Work of 19,689 male colorectal cancer patients in Germany, 2012–2022. *pag. 14*

Table S7: Association between socio-economic position and Return-to-Work of 12,485 female colorectal cancer patients in Germany, 2012–2022. *pag. 15*

Figure S5: Visual check of model assumptions for the adjusted logistic regressions of 'Successful Initial RTW' among 19,689 male colorectal cancer patients in Germany, 2012–2022. *pag. 16*

Figure S6: Visual check of model assumptions for the adjusted logistic regressions of 'Successful Long−Term RTW' among 15,500 male colorectal cancer patients in Germany, 2012–2022. *pag. 17*

Figure S7: Visual check of model assumptions for the adjusted logistic regressions of 'Successful Initial RTW' among 12,485 female colorectal cancer patients in Germany, 2012–2022. *pag. 18*

Figure S8: Visual check of model assumptions for the adjusted logistic regressions of 'Successful Long−Term RTW' among 10,099 female colorectal cancer patients in Germany, 2012–2022. *pag. 19*

Supplementary Methods

Detailed information on the setting

The German national statutory pension insurance offers extensive outpatient or inpatient rehabilitation services to oncological patients. Following hospitalisation, the statutory medical rehabilitation is typically provided in a specialised rehabilitation clinic as inpatient treatment for 3 to 4 weeks [1], with the aim of improving the quality of life, avoiding or reducing permanent physical and mental impairments and maintaining or regaining the ability to work. This medical (follow-up) rehabilitation can be supplemented by vocational rehabilitation, which either initiates the RTW through a gradual re-entry or supports occupational retraining for people who are no longer able to practice their former occupation due to their health condition. In addition, people have the opportunity to apply for inpatient rehabilitation through their general practitioner or therapist later in the rehabilitation process. Within a one to two-year period, oncological rehabilitation can be repeated if special requirements are met. For the majority of the working population, the rehabilitation costs are covered by the German Pension Insurance. At the end of 2023, 58.49 million people without a pension were actively insured in the statutory pension insurance or were covered through an insured family member [2]. In 2024, 87.2% of the working population in Germany were covered by statutory pension insurance [3]. Employees with temporary or fixed-term contracts are subject to compulsory statutory pension insurance and are therefore included in the database. Groups whose rehabilitation is typically not covered by the pension insurance include, for example, civil servants, self-employed or occupational groups with professional representation such as physicians and lawyers. In 2024, this group comprised around 4.13 million individuals (89% of all self-employed persons) [4].

References

1. Gerdes N, Zwingmann C, Jäckel W. The system of rehabilitation in Germany. In: Research in Rehabilitation: Results from a Research Network in Southwest Germany. Stuttgart: Schattauer; 2006. p. 3–19.

2. Deutsche Rentenversicherung Bund. Versichertenbericht 2025 [Internet]. Berlin: Deutsche Rentenversicherung Bund, Grundsatz- und Querschnittsbereich: Forschung und Entwicklung, Dezernat 0630 - Statistische Analysen; 2025 [cited 2025 May 27]. Available from: https://www.deutsche-rentenversicherung.de/SharedDocs/Downloads/DE/Statistiken-und-Berichte/Berichte/versichertenbericht_2025.pdf

3. Statistisches Bundesamt (Destatis). Statistisches Bundesamt [Internet]. 2025 [cited 2025 Jul 25]. Gesetzlich Rentenversicherte. Available from: https://www.destatis.de/DE/Themen/Arbeit/Arbeitsmarkt/Qualitaet-Arbeit/Dimension-4/gesetzlich-rentenversichertel.html

4. Deutsche Rentenversicherung Bund. Selbstständige in die gesetzliche Rentenversicherung einbeziehen. rentenupdate [Internet]. 2026 [cited 2026 Apr 7]. Available from: https://rentenupdate.drv-bund.de/DE/1_Archiv/Archiv/2026/12_Selbststaendige.html

Supplementary Figures and Tables

Table S1: Income thresholds for low, medium, and high income for the years 2011–2020.

Year	n	33rd Percentile of Income	67th Percentile of Income
2011	3,041	€22,386.33	€36,575.00
2012	3,053	€24,314.00	€37,757.33
2013	3,221	€24,507.00	€38,861.00
2014	3,213	€24,642.00	€40,362.33
2015	3,415	€25,284.00	€40,618.00
2016	3,769	€25,778.00	€41,710.00
2017	3,782	€26,848.33	€43,820.00
2018	2,967	€26,940.00	€43,458.00
2019	3,183	€27,537.00	€44,458.33
2020	2,530	€28,511.00	€45,500.00

The table shows the income tertiles for the year two years prior to the calendar year of medical rehabilitation for all patients in the study sample. This income was subject to social insurance contributions. The inclusion period was 2012–2022.

Data source: FDZ-RV — OSV.RSDV.2020–2022

Table S2: Description of RTW-relevant states in German Pension Insurance data.

State	Description
Employment	Contributory employment subject to social security contributions (*sozialversicherungspflichtige Beschäftigung*), including full-time and part-time work. Also includes self-employment subject to compulsory insurance (*pflichtversicherte Selbständige*) and periods of voluntary contributions (*freiwillige Beiträge*). Midi-jobs (up to €1,300/month in 2022) were classified as employment; mini-jobs (up to €450/month from 2013, rising to €520/month from 2022) were excluded as non-contributory. Information is based on monthly contribution periods recorded in the German Pension Insurance account.
Sick leave	Work incapacity beyond the statutory employer-paid sick pay period. In Germany, employers continue to pay full wages during the first six weeks of illness (*Entgeltfortzahlung*); this period is subject to social security contributions and is therefore classified as employment. After six weeks, or during inpatient treatment, statutory sickness benefit (*Krankengeld*) is paid by the statutory health insurance (approx. 70% of gross income, up to 90% of net income) for a maximum of 78 weeks. This state also includes gradual return-to-work programmes (*stufenweise Wiedereingliederung*), which remain funded by the health insurance provider rather than the employer and are therefore not classified as employment.
Unemployment benefit	Receipt of contributory unemployment benefit (*Arbeitslosengeld I*) from the Federal Employment Agency, paid to persons who meet the eligibility criteria (minimum contribution period, active job-seeking registration).
Unemployment	Neither contributory employment nor any social insurance benefit or pension payment recorded. Includes persons financially supported outside the formal social insurance system, e.g. through a working partner or family.
Disability pension	Reduced earning capacity pension (*Erwerbsminderungsrente*) paid by the German Pension Insurance to individuals whose capacity to work is permanently or long-term reduced due to illness or disability. Eligibility requires that the statutory retirement age has not yet been reached, that at least five years of statutory pension insurance contributions have been accrued including a minimum of three years of contributory employment, and that the ability to work could not be restored through medical or vocational rehabilitation. Disability pensions are generally granted for a fixed term and reviewed periodically. A permanent, indefinite pension is only approved if long-term restoration of work capacity is considered unlikely and the individual is able to work for fewer than three hours per day.
Old-age pension	Statutory retirement pension (*Altersrente*) received upon reaching the applicable statutory retirement age, which varies by birth cohort: 65 years for cohorts born before 1947, rising incrementally to 67 years for cohorts born from 1964 onwards. Early retirement is possible before the statutory age, subject to a permanent reduction in pension payments (*Rentenabschläge*). Under specific conditions, such as a particularly long contributory working life (*besonders langjährig Versicherte*), early old-age pension without deductions may also be claimed. Constitutes the end of working life within the observation framework and is treated as an absorbing state in the model.
Death	Death of the insured person as recorded in the German Pension Insurance database.

The table provides a brief description of each RTW-relevant state in the German Pension Insurance data. The German terms relating to social legislation are included in italics for further reference.

Figure S1: Simplified directed acyclic graph.

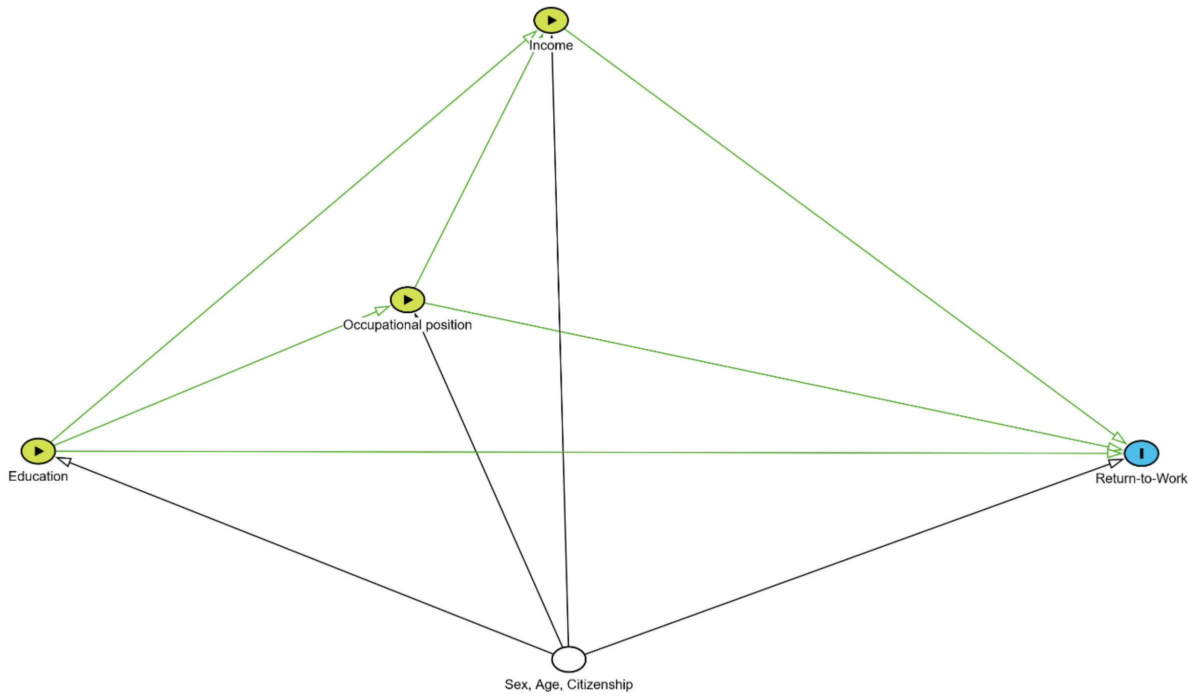

The minimum adjustment set for confounding between socioeconomic indicators (education, occupational position, income) and the return to work was identified in a directed acyclic graph, using the DAGitty program.
ALT TEXT: A graph that illustrates the assumption that education, occupational position and income influence the return to work and that gender, age and citizenship confound this effect.

Table S3: Restricted mean survival time in months of 32,174 colorectal cancer patients in Germany, 2013–2022.

**Restricted mean survival time**	**Employment**	**Sick leave**	**Unemployment**	**Unemployment benefit**	**Disability pension**	**Old-age pension**	**Death**
Education							
Primary or secondary (ISCED-2011: 1–3)	26	3	2	4	11	9	5
Post-secondary (ISCED-2011: 4)	29	3	2	2	10	8	6
Tertiary (ISCED-2011: 5–8)	34	2	3	2	5	8	5
Unknown	24	3	2	4	11	10	7
**Occupational position**							
Unskilled, manual	26	3	2	3	11	9	6
Skilled, manual	28	3	2	3	9	9	6
Unskilled, non-manual	26	3	2	3	11	9	5
Skilled, non-manual	31	3	2	2	9	8	5
Highly skilled	34	2	3	2	6	7	5
**Income**							
Low	24	3	2	4	13	9	6
Middle	29	3	1	2	10	9	6
High	34	3	3	2	6	8	5
**Total**							
Total	29	3	2	3	10	8	6

The supplementary table displays the restricted mean time in months of the five years following medical rehabilitation presented in Figure 3.

Abbreviations: ISCED 2011, International Standard Classification of Education — 2011 version.

Data source: FDZ-RV — OSV.RSDV.2020–2022

Figure S2: Comparison of the probabilities of employment, unemployment, unemployment benefit, sick leave, disability pension, old-age pension and death after medical rehabilitation, stratified by income (a), occupational position (b) and education (c) among 19,689 male and 12,485 female patients with colorectal cancer in Germany, 2013–2022.

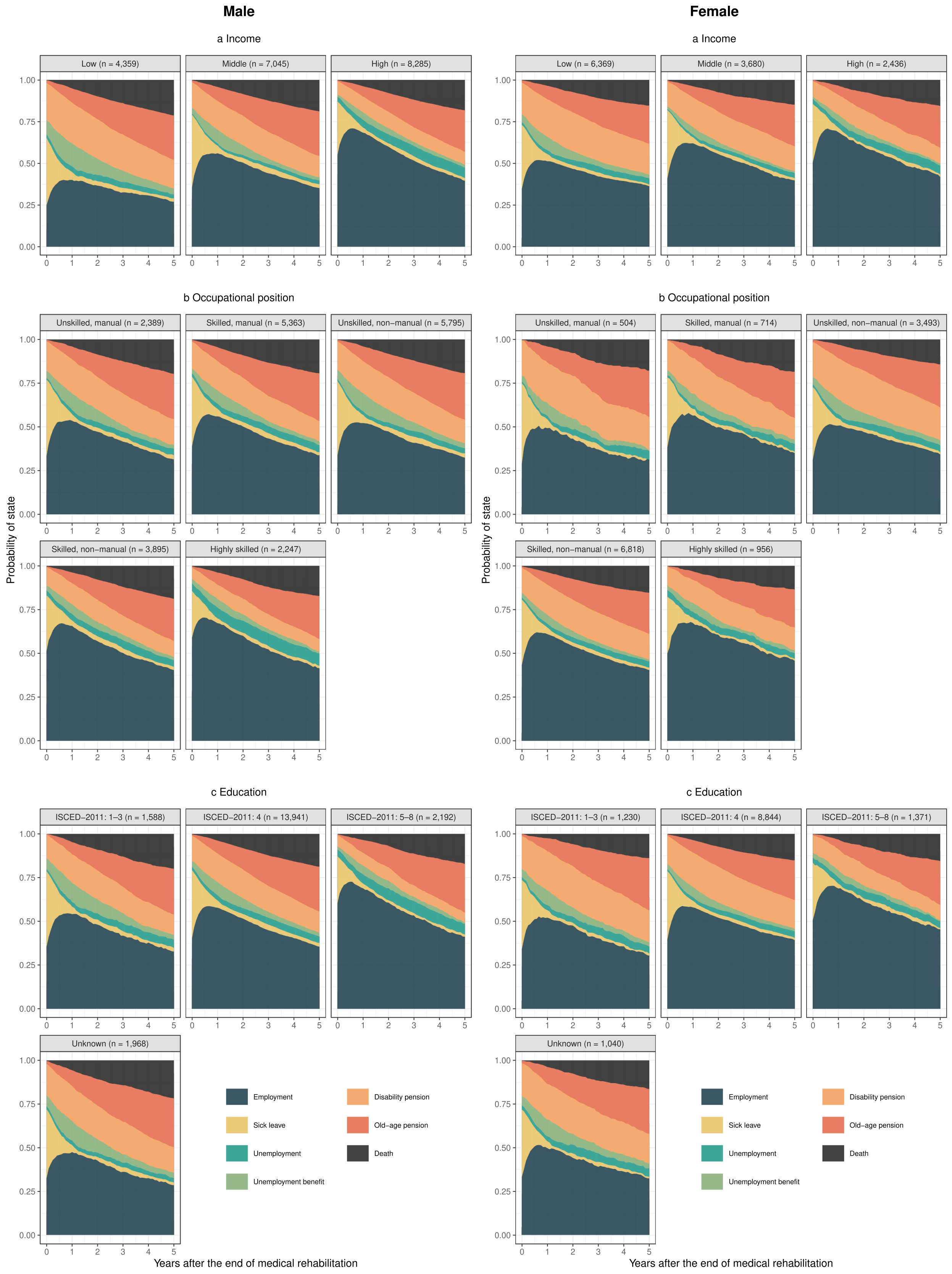

The figure shows the differences in the estimated average number of months for the states "Employment", "Unemployment", "Unemployment Benefit", "Sick leave", "Disability pension", "Old-age pension", and "Death" over a period of five years after the end of medical rehabilitation, stratified by sex. Table S4 and Table S5 the sex-specific restricted mean survival time for each state in months over the observation period of five years.

Abbreviations: ISCED 2011, International Standard Classification of Education — 2011 version

Data source: FDZ-RV — OSV.RSDV.2020–2022

ALT TEXT: Graphs comparing the probabilities of being in a state for different levels of the socio-economic indicators for male and female patients, with higher probabilities of being employed and lower probabilities of being unemployed or in disability pension for patients with a higher socio-economic position for both groups.

Table S4: Restricted mean survival time in months of 19,689 male colorectal cancer patients in Germany, 2013–2022.

Restricted mean survival time	Employment	Sick leave	Unemployment	Unemployment benefit	Disability pension	Old-age pension	Death
**Education**							
Primary or secondary (ISCED-2011: 1–3)	27	3	2	4	9	8	6
Post-secondary (ISCED-2011: 4)	29	3	2	3	9	8	6
Tertiary (ISCED-2011: 5–8)	34	2	4	2	4	9	5
Unknown	23	3	2	4	11	10	7
**Occupational position**							
Unskilled, manual	26	3	2	3	11	9	6
Skilled, manual	28	3	2	3	9	9	6
Unskilled, non-manual	26	3	2	4	10	9	6
Skilled, non-manual	32	3	2	2	7	8	6
Highly skilled	34	2	4	2	5	8	6
**Income**							
Low	21	3	2	5	13	9	7
Middle	28	4	1	3	10	9	6
High	34	3	3	2	5	8	6
**Total**							
Total	29	3	2	3	9	9	6

The supplementary table displays the restricted mean time in months of the five years following medical rehabilitation for male rehabilitands with colorectal cancer presented in Figure S2.

Abbreviations: ISCED 2011, International Standard Classification of Education — 2011 version.

Data source: FDZ-RV — OSV.RSDV.2020–2022

Table S5: Restricted mean survival time in months of 12,485 female colorectal cancer patients in Germany, 2013–2022.

Restricted mean survival time	Employment	Sick leave	Unemployment	Unemployment benefit	Disability pension	Old-age pension	Death
**Education**							
Primary or secondary (ISCED-2011: 1–3)	25	3	2	3	13	9	4
Post-secondary (ISCED-2011: 4)	29	3	2	2	11	8	5
Tertiary (ISCED-2011: 5–8)	35	3	2	2	6	7	5
Unknown	25	3	2	4	12	9	5
**Occupational position**							
Unskilled, manual	24	3	2	3	13	9	6
Skilled, manual	28	3	2	2	11	8	6
Unskilled, non-manual	26	3	2	3	13	8	5
Skilled, non-manual	31	3	2	2	10	8	5
Highly skilled	34	2	2	2	8	7	5
**Income**							
Low	26	3	2	3	13	8	5
Middle	31	3	1	2	10	8	5
High	34	2	3	2	7	8	5
**Total**							
Total	29	3	2	2	11	8	5

The supplementary table displays the restricted mean time in months of the five years following medical rehabilitation for female rehabilitands with colorectal cancer presented in Figure S2.

Abbreviations: ISCED 2011, International Standard Classification of Education — 2011 version.

Data source: FDZ-RV — OSV.RSDV.2020–2022

Figure S3: Visual check of model assumptions for the adjusted logistic regressions of 'Successful Initial RTW' among 32,174 colorectal cancer patients in Germany, 2012–2022.

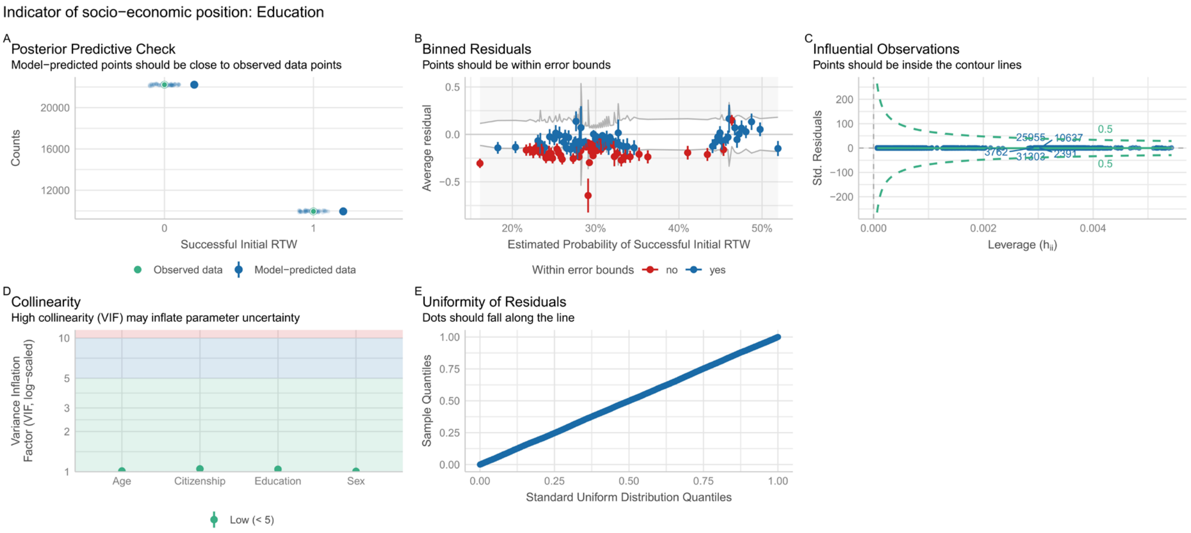

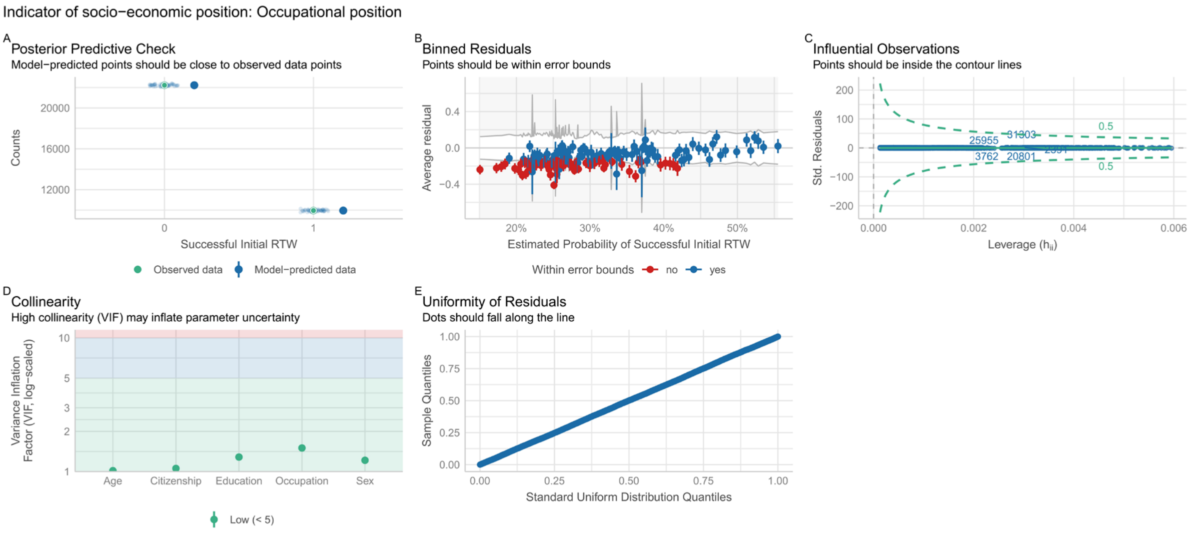

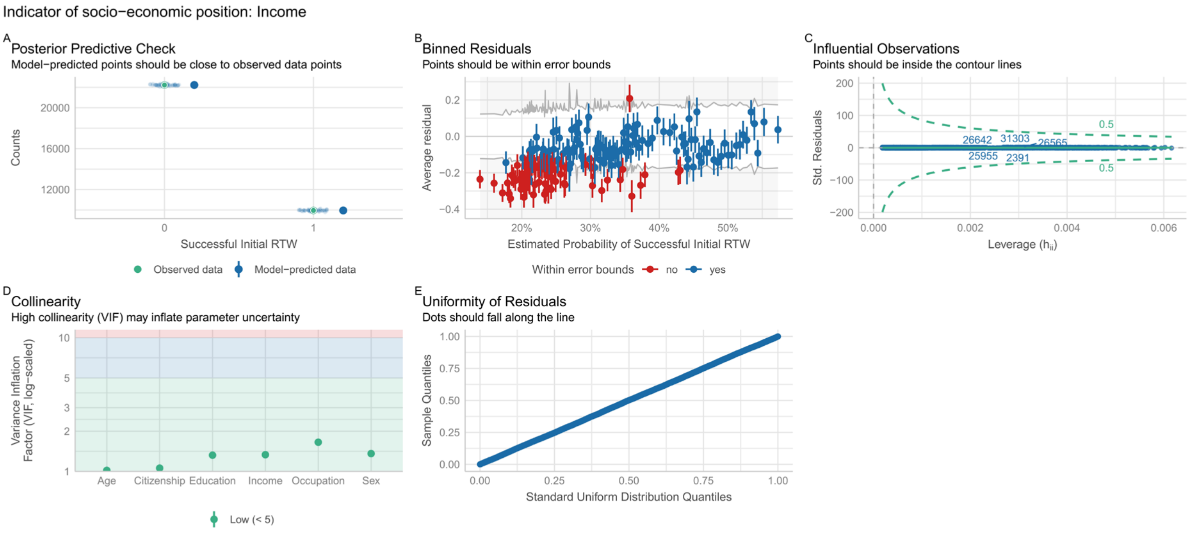

The plots visually check the various model assumptions for the 'Initial RTW' results presented in Table 3.
Abbreviations: RTW, Return-to-Work.
Data source: FDZ-RV — OSV.RSDV.2020–2022

Figure S4: Visual check of model assumptions for the adjusted logistic regressions of 'Successful Long–Term RTW' among 25,599 colorectal cancer patients in Germany, 2012–2022.

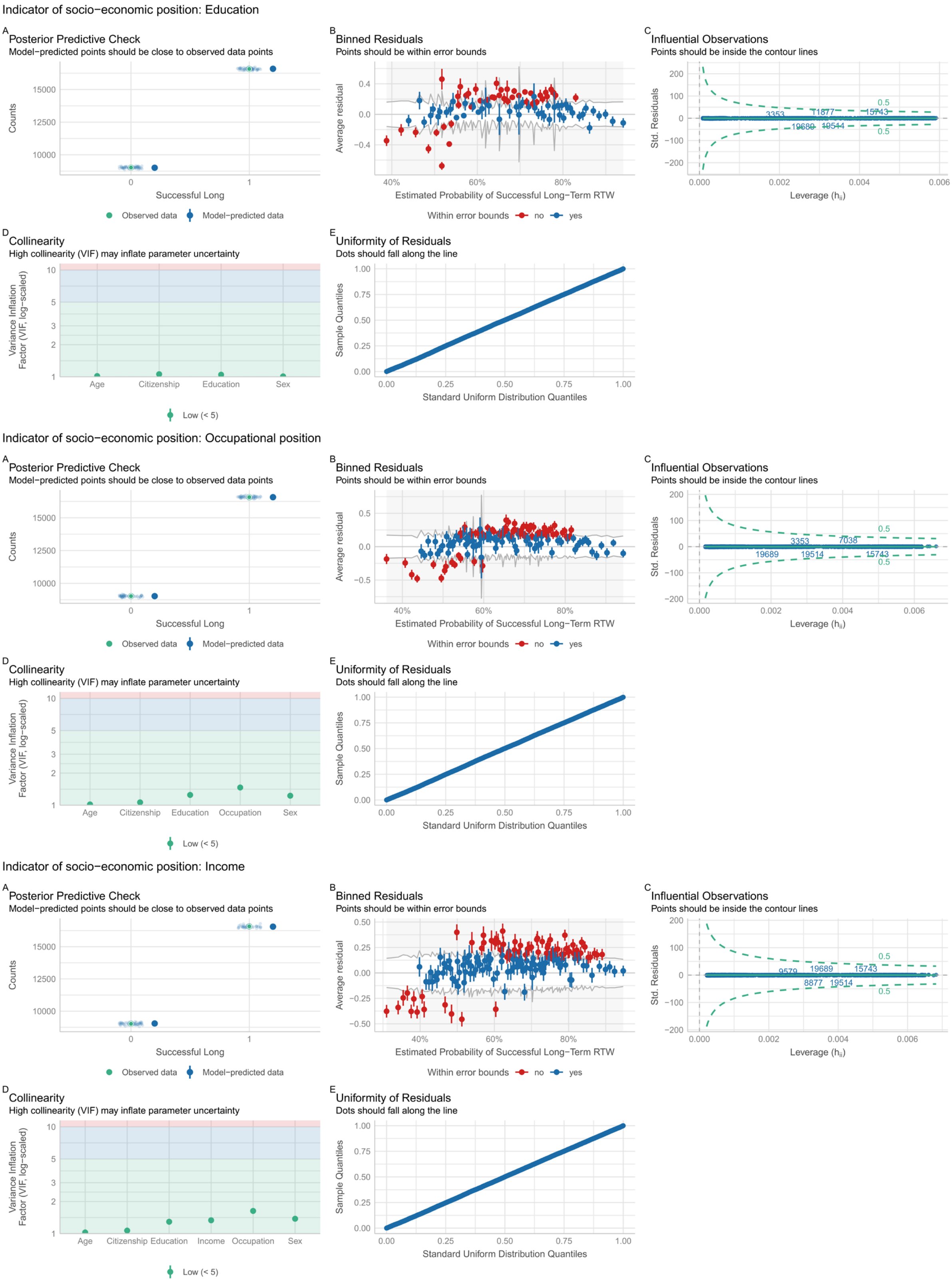

The plots visually check the various model assumptions for the 'Long-Term RTW' results presented in Table 3.

Abbreviations: RTW, Return-to-Work.

Data source: FDZ-RV — OSV.RSDV.2020–2022

Table S6: Association between socio-economic position and Return-to-Work of 19,689 male colorectal cancer patients in Germany, 2012–2022.

	Initial RTW			Long-Term RTW		
**Indicators of socio-economic position**	**Event/n**	**OR (95% CI)**	**aOR (95% CI)**	**Event/n**	**OR (95% CI)**	**aOR (95% CI)**
**Education**[a]						
Primary or secondary (ISCED-2011: 1–3)	402/1,588	—	—	784/1,251	—	—
Post-secondary (ISCED-2011: 4)	4,220/13,941	1.28 (1.14 to 1.44)	1.21 (1.08 to 1.37)	7,204/11,046	1.12 (0.99 to 1.26)	1.11 (0.98 to 1.26)
Tertiary (ISCED-2011: 5–8)	1,094/2,192	2.94 (2.56 to 3.39)	2.77 (2.40 to 3.20)	1,325/1,666	2.31 (1.96 to 2.73)	2.31 (1.95 to 2.75)
Unknown	468/1,968	0.92 (0.79 to 1.07)	0.90 (0.77 to 1.05)	813/1,537	0.67 (0.57 to 0.78)	0.67 (0.57 to 0.78)
**Occupational position**[a,b]						
Unskilled, manual	572/2,389	—	—	1,169/1,922	—	—
Skilled, manual	1,526/5,363	1.26 (1.13 to 1.41)	1.20 (1.07 to 1.34)	2,700/4,296	1.09 (0.98 to 1.22)	1.04 (0.93 to 1.16)
Unskilled, non-manual	1,437/5,795	1.05 (0.94 to 1.17)	1.02 (0.92 to 1.14)	2,692/4,535	0.94 (0.84 to 1.05)	0.91 (0.82 to 1.02)
Skilled, non-manual	1,563/3,895	2.13 (1.90 to 2.39)	1.82 (1.62 to 2.05)	2,223/3,030	1.77 (1.57 to 2.00)	1.50 (1.32 to 1.70)
Highly skilled	1,086/2,247	2.97 (2.62 to 3.37)	2.29 (2.00 to 2.62)	1,342/1,717	2.31 (1.99 to 2.67)	1.77 (1.51 to 2.08)
**Income**[a,b,c]						
Low	817/4,359	—	—	1,585/3,425	—	—
Middle	1,770/7,045	1.45 (1.33 to 1.60)	1.46 (1.33 to 1.61)	3,490/5,528	1.99 (1.82 to 2.17)	2.08 (1.90 to 2.28)
High	3,597/8,285	3.33 (3.05 to 3.63)	2.79 (2.54 to 3.06)	5,051/6,547	3.92 (3.59 to 4.28)	3.72 (3.37 to 4.10)

[a]Adjusted for age and citizenship

[b]Additionally adjusted for education

[c]Additionally adjusted for occupational position

Abbreviations: CI = confidence interval, OR = odds ratio, aOR = adjusted odds ratio, ISCED 2011 = International Standard Classification of Education — 2011 version, RTW = Return-to-Work

The models for Initial RTW are based on the complete study sample of 19,689 male colorectal cancer patients. The models for Long-Term RTW require a minimum observation period of 24 months, during which the statutory retirement age is not reached. These models are therefore based on 15,500 male colorectal cancer patients.

Em dash (—) indicates the reference category.

Data source: FDZ-RV — OSV.RSDV.2020–2022

Table S7: Association between socio-economic position and Return-to-Work of 12,485 female colorectal cancer patients in Germany, 2012–2022.

	Initial RTW			Long-Term RTW		
**Indicators of socio-economic position**	**Event/n**	**OR (95% CI)**	**aOR (95% CI)**	**Event/n**	**OR (95% CI)**	**aOR (95% CI)**
**Education[a]**						
Primary or secondary (ISCED-2011: 1–3)	316/1,230	—	—	577/996	—	—
Post-secondary (ISCED-2011: 4)	2,662/8,844	1.25 (1.09 to 1.43)	1.21 (1.05 to 1.38)	4,589/7,190	1.28 (1.12 to 1.47)	1.16 (1.01 to 1.33)
Tertiary (ISCED-2011: 5–8)	551/1,371	1.94 (1.64 to 2.30)	1.87 (1.58 to 2.22)	818/1,076	2.30 (1.91 to 2.78)	2.07 (1.71 to 2.51)
Unknown	239/1,040	0.86 (0.71 to 1.05)	0.85 (0.70 to 1.03)	465/837	0.91 (0.75 to 1.09)	0.86 (0.71 to 1.04)
**Occupational position[a,b]**						
Unskilled, manual	112/504	—	—	230/425	—	—
Skilled, manual	195/714	1.32 (1.01 to 1.72)	1.27 (0.97 to 1.66)	363/597	1.32 (1.02 to 1.69)	1.18 (0.91 to 1.53)
Unskilled, non-manual	799/3,493	1.04 (0.83 to 1.30)	1.03 (0.82 to 1.29)	1,590/2,795	1.12 (0.91 to 1.37)	1.05 (0.85 to 1.30)
Skilled, non-manual	2,290/6,818	1.77 (1.43 to 2.21)	1.64 (1.32 to 2.05)	3,689/5,485	1.74 (1.43 to 2.12)	1.47 (1.20 to 1.81)
Highly skilled	372/956	2.23 (1.75 to 2.86)	1.90 (1.48 to 2.46)	577/797	2.22 (1.74 to 2.85)	1.62 (1.25 to 2.10)
**Income[a,b,c]**						
Low	1,702/6,369	—	—	2,935/5,131	—	—
Middle	1,115/3,680	1.19 (1.09 to 1.30)	1.07 (0.97 to 1.17)	2,014/2,992	1.54 (1.40 to 1.69)	1.52 (1.38 to 1.68)
High	951/2,436	1.76 (1.59 to 1.94)	1.40 (1.26 to 1.56)	1,500/1,976	2.36 (2.10 to 2.65)	2.22 (1.95 to 2.53)

[a]Adjusted for age and citizenship

[b]Additionally adjusted for education

[c]Additionally adjusted for occupational position

Abbreviations: CI = confidence interval, OR = odds ratio, aOR = adjusted odds ratio, ISCED 2011 = International Standard Classification of Education — 2011 version, RTW = Return-to-Work

The models for Initial RTW are based on the complete study sample of 12,485 female colorectal cancer patients. The models for Long-Term RTW require a minimum observation period of 24 months, during which the statutory retirement age is not reached. These models are therefore based on 10,099 female colorectal cancer patients.

Em dash (—) indicates the reference category.

Data source: FDZ-RV — OSV.RSDV.2020–2022

Figure S5: Visual check of model assumptions for the adjusted logistic regressions of 'Successful Initial RTW' among 19,689 male colorectal cancer patients in Germany, 2012–2022.

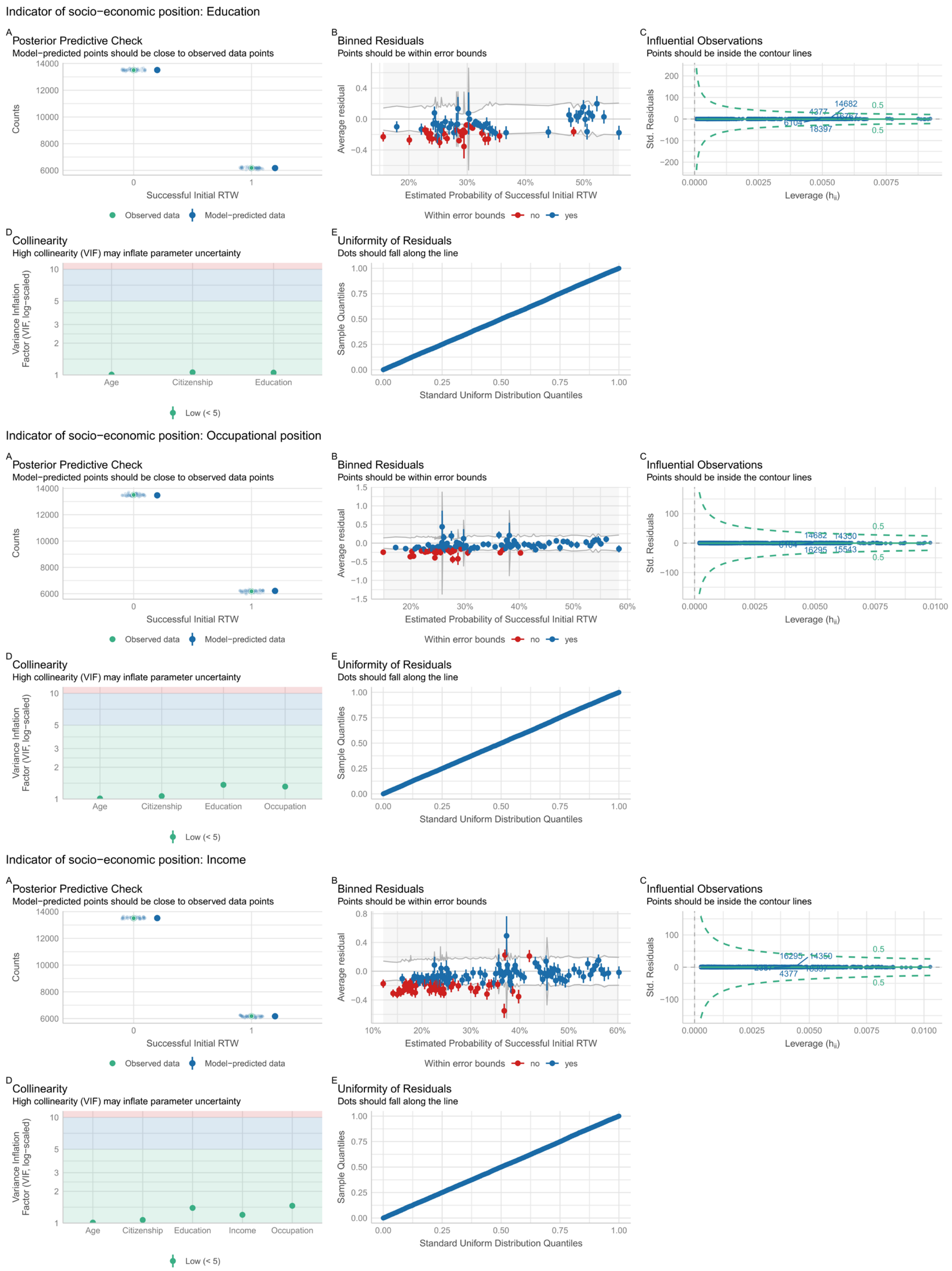

The plots visually check the various model assumptions for the 'Initial RTW' results presented in Table S6.
Abbreviations: RTW, Return-to-Work.
Data source: FDZ-RV — OSV.RSDV.2020–2022

Figure S6: Visual check of model assumptions for the adjusted logistic regressions of 'Successful Long–Term RTW' among 15,500 male colorectal cancer patients in Germany, 2012–2022.

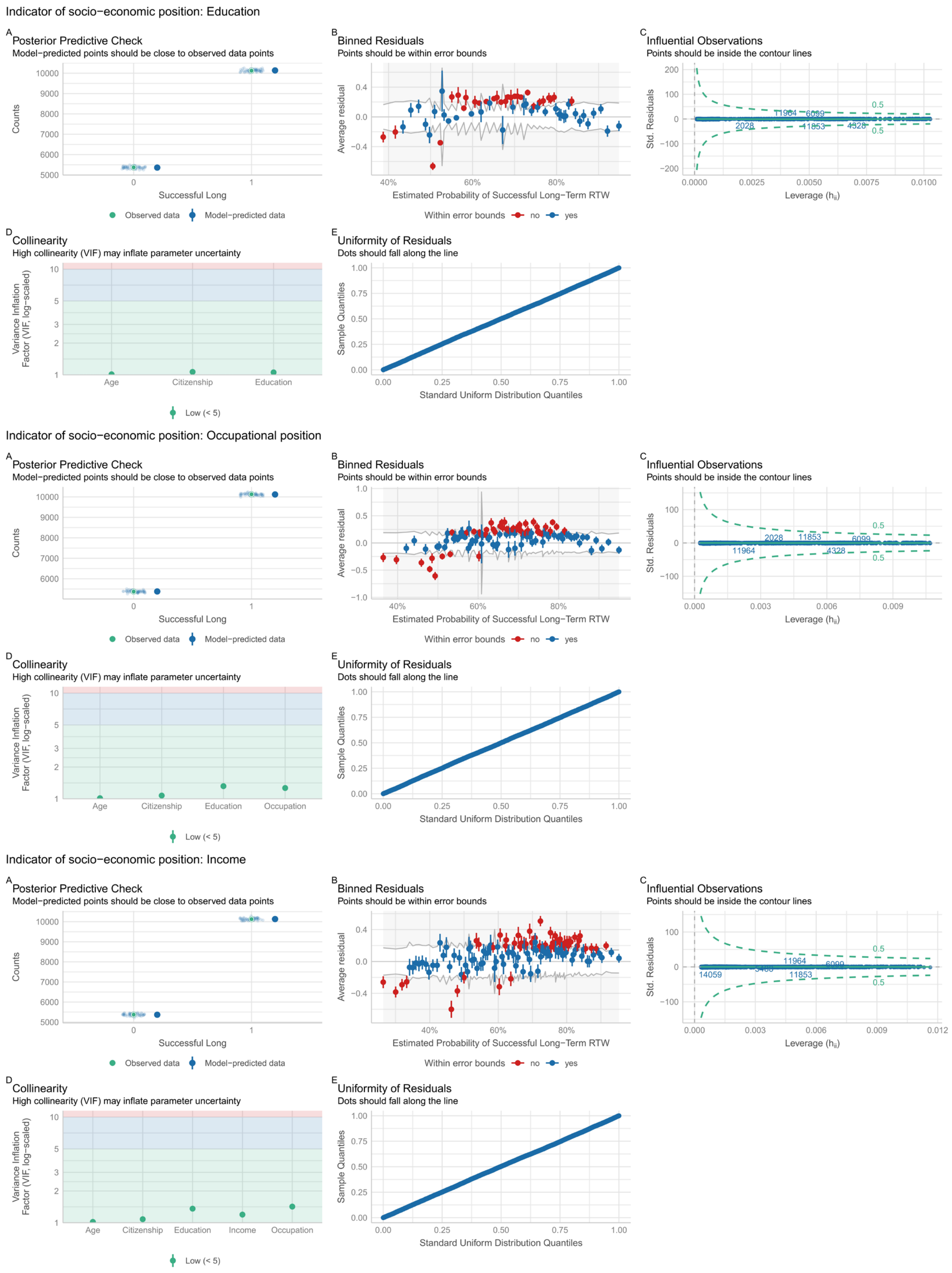

The plots visually check the various model assumptions for the 'Long-Term RTW' results presented in Table S6.

Abbreviations: RTW, Return-to-Work.

Data source: FDZ-RV — OSV.RSDV.2020–2022

Figure S7: Visual check of model assumptions for the adjusted logistic regressions of 'Successful Initial RTW' among 12,485 female colorectal cancer patients in Germany, 2012–2022.

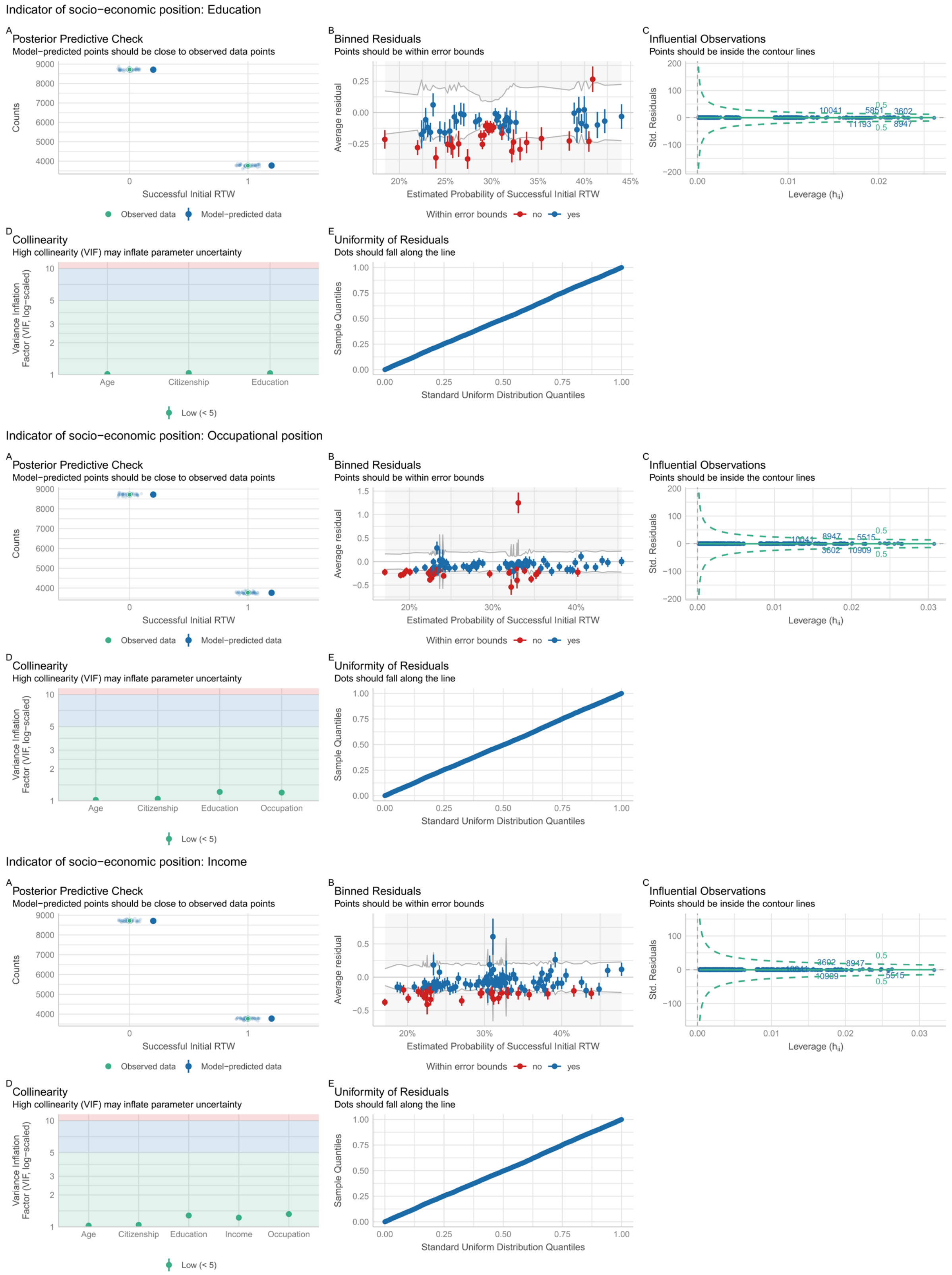

The plots visually check the various model assumptions for the 'Initial RTW' results presented in Table S7.
Abbreviations: RTW, Return-to-Work.
Data source: FDZ-RV — OSV.RSDV.2020–2022

Figure S8: Visual check of model assumptions for the adjusted logistic regressions of 'Successful Long–Term RTW' among 10,099 female colorectal cancer patients in Germany, 2012–2022.

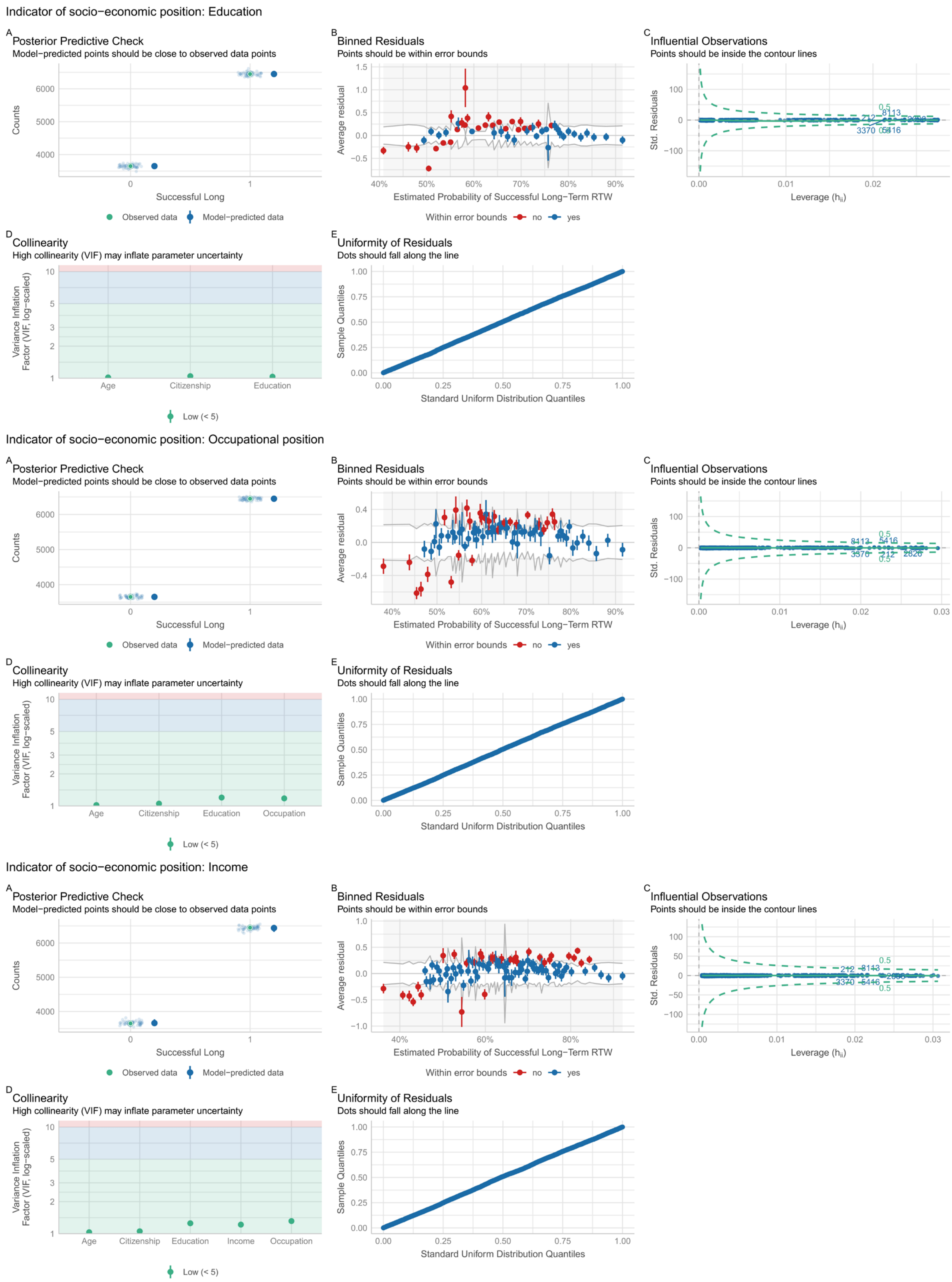

The plots visually check the various model assumptions for the 'Long-Term RTW' results presented in Table S7.

Abbreviations: RTW, Return-to-Work.

Data source: FDZ-RV — OSV.RSDV.2020–2022
